# Medulloblastoma in an Adult With Late Extraneural Metastases to the Mediastinum

**DOI:** 10.1177/2324709614532798

**Published:** 2014-04-17

**Authors:** Abhimanyu Ghose, John C. Morris, John C. Breneman, James Essell, Jiang Wang, Sadia Benzaquen

**Affiliations:** 1 University of Cincinnati, Cincinnati, OH, USA; 2 Oncology Hematology Care Inc, Cincinnati, OH, USA

**Keywords:** medulloblastoma, metastasis, adult, mediastinum, extraneural

## Abstract

*Background*. Medulloblastoma, although the most common brain tumor of childhood, is exceedingly rare in adults. These tumors have a propensity for local recurrence and to metastasize along the leptomeninges; however, extraneural metastases are very rare and typically occur in the bone or bone marrow. We have not come across any case in literature of medulloblastoma with mediastinal metastases in an adult. *Case Presentation*. We report a case of medulloblastoma in a 38-year-old lady who was treated with surgery followed by craniospinal radiation. Ten years later she presented with hoarseness from true vocal cord paralysis. She was diagnosed to have infiltrating metastases of her medulloblastoma to the mediastinum, which was confirmed by biopsy. There was no local recurrence. This was treated with chemotherapy followed by stem cell rescue, and she remained progression free for 2 years. *Conclusion*. Medulloblastomas are rare in adults and can present with late extraneural metastases following treatment. Although most common reported sites are bone and bone marrow, late metastases to other unexpected areas like the mediastinum are possible too and warrant awareness. This can be treated with chemotherapy followed by high-dose chemotherapy and stem cell rescue in a young patient with good performance status.

## Background

Medulloblastomas are most common malignant brain tumors of the pediatric population, typically arising in the cerebellum. Annually around 500 children are diagnosed in the United States with a peak incidence between 5 and 9 years of age.^1^ Medulloblastomas are extremely rare after the fourth decade. They commonly present with symptoms of increased intracranial pressure that may vary depending on the location, with midline tumors causing truncal ataxia, while those in the lateral cerebellar hemispheres causing incoordination of the extremities. Medulloblastomas have a propensity to recur locally and metastasize along the subarachnoid space, often presenting with drop metastasis along the spinal cord. Extraneural metastases are exceedingly rare with the most common reported sites being bone and bone marrow.^2^ We report a case of an adult with medulloblastoma, who recurred with extensive metastasis to the mediastinum 10 years after initial successful treatment of the primary tumor.

## Case Presentation

**Figure 1.**
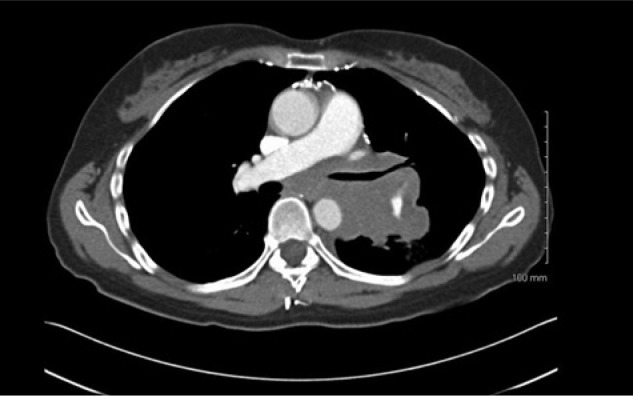
CT scan showing the infiltrating mediastinal mass.

**Figure 2.**
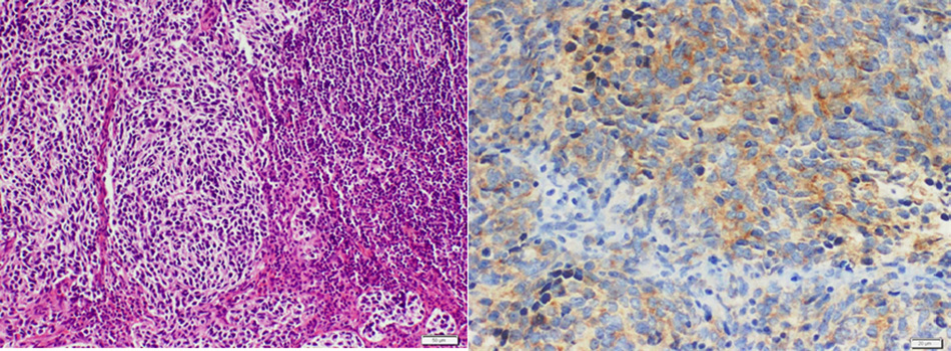
Medulloblastoma metastasized to supraclavicular lymph node. A. H&E: High-grade blue tumor cells forming sheets and nodules. B. Immunohistochemistry: Tumor cells are positive for synaptophysin.

A 38-year-old woman presented to the emergency department complaining of worsening headaches, visual disturbances, nausea, and vomiting, with difficulty in gait and coordination over the previous 4 to 5 months. The problems were worst early in the morning. Examination showed dysmetria, bilateral nystagmus on lateral gaze, normal motor strength, and normal reflexes throughout with no sensory deficits. Visual fields were normal. Magnetic resonance imaging (MRI) showed a large left cerebellar tumor with Chiari 1 malformation and mild hydrocephalus. On further staging, there was no evidence of metastasis. She was begun on corticosteroids and underwent a left suboccipital craniectomy and excision of the tumor. Pathology showed a densely cellular and infiltrative tumor composed of cells with round to oval basophilic, nuclei, and scant pink cytoplasm. It infiltrated to the cortical surface and in several areas was arranged in variably sized nodules. Immunohistochemistry for glial fibrillary acidic protein (GFAP) and neuron-specific enolase (NSE) revealed scattered positive cells. The tumor was consistent with classic medulloblastoma with high MIB1 proliferative activity >10%. She was classified as standard risk for recurrence. Analysis of molecular markers was not done as its role was not established then. Postoperatively she received 36 Gy craniospinal radiation with an 18 Gy boost to the posterior fossa followed by adjuvant chemotherapy with carboplatin and etoposide. She did well for 10 years until she presented with generalized weakness and hoarseness. ENT evaluation demonstrated left true vocal cord paralysis. Computed tomography (CT) showed a 4.2 × 6.8 cm infiltrating mass that surrounded and extrinsically compressed the left main pulmonary artery, left mainstem bronchus, and distal trachea. The mass was inseparable from the aortic arch and involved the left hilar, subcarinal, pretracheal, and right paratracheal nodes (Figure 1). Positron emission tomography (PET)/CT showed ^18^FDG-avid masses involving the left lower lobe, left hilum, and mediastinum, and bilateral supraclavicular lymph nodes. Excisional biopsy of a left supraclavicular lymph node showed metastatic high-grade round blue cell malignant neoplasm positive for GFAP, NSE, and synaptophysin (Figure 2). After comparison to the original biopsy, a diagnosis of metastatic medulloblastoma was rendered. MRI of the head and spine showed no evidence of tumor or metastases in the central nervous system. She was treated with 3 cycles of weekly cisplatin 30 mg/m^2^ and irinotecan 65 mg/m^2^ after which PET scan showed a compete response. This was followed by high-dose carboplatin, thiotepa, and etoposide conditioning followed by autologous stem cell transplant. She tolerated the treatment well and achieved a complete response. Two years later, PET/CT showed increased activity in the mediastinum and supraclavicular lymph nodes. A biopsy confirmed relapse. She was treated with 6 cycles of carboplatin AUC 4 and gemcitabine 1000 mg/m^2^ every 21 days. After showing an initial response to the chemotherapy, PET/CT showed tumor progression in the aforementioned sites. She was treated with 50.4 Gy intensity modulated radiotherapy. The mass showed a partial response that has been stable for 4 months.

## Discussion

Medulloblastomas are malignant brain tumors arising from primitive neuroepithelial cells in the cerebellum. They are the most common malignant brain tumor of childhood with peak incidence between 5 and 9 years and a much smaller peak between 20 and 24 years of age.^3,4^ Seventy percent are diagnosed before 20 years of age and rare after the fourth decade, consistent with their embryonal origin.^3,4^ Since the leptomeninges are involved in 20% to 25% of patients, both spinal MRI and cerebrospinal fluid (CSF) analysis is recommended in all patients, as the results can be discordant.^5^ Multimodality therapy with resection followed by radiation to the primary site and the craniospinal axis, and systemic chemotherapy is standard.^6^ Treatment of adults is modeled on protocols used in children as there are no prospective studies in adults owing to the rarity of the tumor.

In one series by Pobereskin and Treip on 12 adult patients, all but one—who had bone metastases—experienced either a local recurrence or craniospinal metastases.^7^ In another study by Ang et al on 25 patients, they had either a local recurrence or metastases within the neuraxis.^8^ In a third series of 14 adult patients, the recurrence was primarily in the central nervous system (CNS) apart from bone or bone marrow metastases observed in 3 patients.^9^ Similarly, in another series of 42 patients by Prados et al, there were no extraneural metastases apart from 2 patients who developed bone lesions.^10^ Extraneural metastases are exceedingly rare in literature. We came across one case with widespread metastatic disease and local recurrence that recurred in the pelvic lymph nodes and soft tissues.^11^ We have not come across any adult medulloblastoma patient with mediastinal metastases like our patient.

Since spinal leptomeninges can be involved in 20% to 25% of patients, both MRI spine and CSF analysis is recommended in all patients at diagnosis, as the results can be discordant.^5^ Multimodality therapy with maximal safe resection followed by radiation to the primary site and the craniospinal axis and systemic chemotherapy is the standard treatment in children. Neurologic and endocrine complications are limiting factors for higher radiation therapy doses, especially in children less than 3 years old. For children more than 3 years of age with complete resection of tumor, 23.4 Gy craniospinal irradiation followed by a posterior fossa boost of 32.4 Gy is the standard recommendation.^6^ Treatment in adults is modeled on protocols in older children as there are no randomized prospective studies in this age group owing to the rarity in this population. In adults with no evidence of residual disease postsurgery or distant metastases, the role of chemotherapy is controversial as they can tolerate higher doses of radiation therapy.

Late relapses are more frequent in adults compared to children.^2,12,13^ There is no standard treatment for adult patients who suffer a relapse. Options include surgical resection where possible, followed by chemotherapy and/or re-irradiation and high-dose chemotherapy followed by stem cell rescue. Multiple case series suggest that re-resection is associated with prolonged survival.^14^ Re-irradiation especially as fractionated stereotactic radiotherapy or radiosurgery seems to result in good local control rates with 65% 1-year survival in one study.^15^ But the cumulative toxicity from re-irradiation especially brain radionecrosis can be a limiting factor. Conventional chemotherapy usually leads to a median survival of 5 months only and there is no large study to suggest the best regimen.^16^ Cisplatin or carboplatin have response rates ranging from 41% to 79% as second- or third-line therapy.^17^ BCNU (carmustine) has a response rate of 33%.^18^ Because the response has been transient with conventional chemotherapy, high-dose therapy with thiotepa, BCNU, cyclophosphamide, carboplatin, and etoposide, both alone and in combination, have been studied. Overall response rates varied from 30% to 60%, but again long-term effects and survival were limited.^19^ With temozolamide some case reports have shown progression-free interval of 8.4 months to a year.^20,21^ Chemotherapy options as suggested by NCCN include high-dose cyclophosphamide ± etoposide, platinum agents/etopside/cyclophosphamide in patients without prior chemotherapy or high-dose cyclophosphamide ± etoposide, oral etopside, temozolamide in patients with prior chemotherapy.^22^ Multiple small studies have suggested a potential role for high-dose chemotherapy followed by stem cell rescue in patients with no evidence of disease after surgical resection or initial chemotherapy.^23-26^ In a study by Gill et al on 10 patients the overall survival was significantly better than a historical compared group of 3.47 years versus 2 years.^24^ Among the various conditioning regimens carboplatin/thiotepa/etoposide has been commonly used.^23^ With this regimen, the median overall survival in 2 studies by Dunkel et al^23^ and Abrey et al^27^ with 10 or more patients has been 12.3 months (range = 0.9-31.3 months) and 34 months (range = 1-63 months), respectively. The treatment-related mortality was 25% and 18%, respectively. Although our patient tolerated the treatment well with a progression-free survival of 2 years, it should be noted that high-dose chemotherapy with stem cell transplantation is not without serious side effects that include myelosuppression, mucositis, diarrhea, nausea, vomiting, and skin toxicities.^26^ In the review by Kostaras and Easaw, out of 66 patients in literature who received stem cell transplantation, 7 suffered from treatment-related mortality. Therefore, careful patient selection is essential for this procedure.^26^

## Conclusion

Medulloblastomas are exceedingly rare in adults. They can present with late extraneural metastases to uncommon sites apart from bone or bone marrow like mediastinum and pelvis in the absence of local or CNS recurrence. Longer follow-up and awareness among physicians is necessary for this kind of presentation. Although the treatment in adults is modeled on pediatric protocols, there are differences as adults can tolerate higher doses of radiation. The role of adjuvant chemotherapy is controversial in adults. In the relapsed setting, high-dose chemotherapy followed by autologous stem cell rescue is a potential option in healthy young patients with no evidence of disease after re-resection or initial chemotherapy. Large studies are lacking on the best conditioning regimen, but small studies suggest carboplatin/thiotepa/etoposide as a good regimen with better long-term survival compared to historical controls.
